# BiomeNet: a database for construction and analysis of functional interaction networks for any species with a sequenced genome

**DOI:** 10.1093/bioinformatics/btz776

**Published:** 2019-10-10

**Authors:** Eiru Kim, Dasom Bae, Sunmo Yang, Gunhwan Ko, Sungho Lee, Byungwook Lee, Insuk Lee

**Affiliations:** 1 Department of Biotechnology, Yonsei University, Seodaemun-gu, Seoul 03722, Korea; 2 Korean Bioinformation Center, KRIBB, Yuseong-gu, Daejeon 34141, Korea

## Abstract

**Motivation:**

Owing to advanced DNA sequencing and genome assembly technology, the number of species with sequenced genomes is rapidly increasing. The aim of the recently launched Earth BioGenome Project is to sequence genomes of all eukaryotic species on Earth over the next 10 years, making it feasible to obtain genomic blueprints of the majority of animal and plant species by this time. Genetic models of the sequenced species will later be subject to functional annotation, and a comprehensive molecular network should facilitate functional analysis of individual genes and pathways. However, network databases are lagging behind genome sequencing projects as even the largest network database provides gene networks for less than 10% of sequenced eukaryotic genomes, and the knowledge gap between genomes and interactomes continues to widen.

**Results:**

We present BiomeNet, a database of 95 scored networks comprising over 8 million co-functional links, which can build and analyze gene networks for any species with the sequenced genome. BiomeNet transfers functional interactions between orthologous proteins from source networks to the target species within minutes and automatically constructs gene networks with the quality comparable to that of existing networks. BiomeNet enables assembly of the first-in-species gene networks not available through other databases, which are highly predictive of diverse biological processes and can also provide network analysis by extracting subnetworks for individual biological processes and network-based gene prioritizations. These data indicate that BiomeNet could enhance the benefits of decoding the genomes of various species, thus improving our understanding of the Earth’ biodiversity.

**Availability and implementation:**

The BiomeNet is freely available at http://kobic.re.kr/biomenet/.

**Supplementary information:**

Supplementary data are available at *Bioinformatics* online.

## 1 Introduction

Advances in DNA sequencing and genome assembly technology promoted rapid increase in the number of species with sequenced genome. By April 2019, the Genome OnLine database (Mukherjee *et al.*, 2019) reported sequenced genomes for more than 134 000 cellular organisms, including over 5000 eukaryotic species. Annotation of every sequenced genome should be followed by functional annotation of individual genes and pathways, and construction of biological networks significantly facilitates functional analysis of genomes by disclosing interactions among different genes. However, creation of network databases has been lagging far behind genome projects and currently, the largest network database, STRING v11 (Szklarczyk *et al.*, 2019), provides gene networks for not more than 500 eukaryotic species, which is less than 10% of all sequenced eukaryote genomes. Considering a recent launch of the Earth BioGenome Project (Lewin *et al.*, 2018) aiming to sequence genomes of all eukaryotic species on Earth in the next 10 years, it can be expected that the knowledge gap between genomes and interactomes will continue to widen. This problem may be solved by public computational pipelines that can automatically construct gene network models for every sequenced genome.

**Fig. 1.**
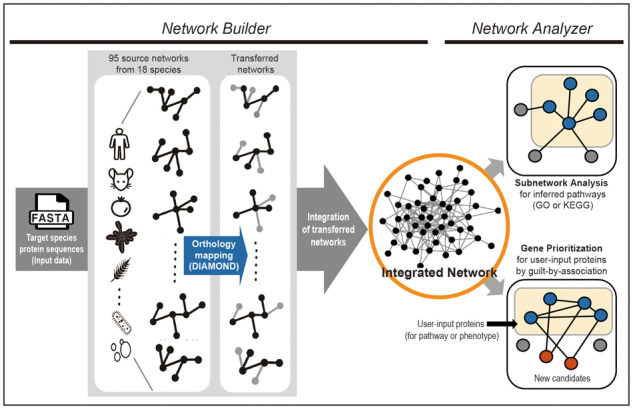
Overview of network building and analysis processes provided by the BiomeNet web server

Here, we present BiomeNet (http://kobic.re.kr/biomenet/), a database that enables construction and analysis of gene networks for any sequenced genome in the Earth’s biome. The workflow of BiomeNet is summarized in Figure 1. Users can submit protein sequences of target species in the FASTA format and the BiomeNet ‘Network Builder’ server extracts functional interactions between orthologous genes from 95 source networks comprising ∼8 million links, which have been scored and evaluated in 18 species (five animals, six plants, five bacteria and two fungi). By using distributed computing and a fast homology search algorithm, BiomeNet can return a newly constructed gene network for most species in a few minutes. Furthermore, the BiomeNet ‘Network Analyzer’ server provides two network-based tools for functional analysis of the obtained network: (i) subnetwork extraction for individual inferred Gene Ontology biological processes (GO-BP) (The Gene Ontology, 2017) and KEGG pathways (Kanehisa *et al.*, 2017) and (ii) network-based gene prioritization for the user-input gene set (Wang and Marcotte, 2010).

We assessed the networks automatically constructed by BiomeNet for various animal and plant species for prediction of biological process annotations by AgriGO v2 (Tian *et al.*, 2017) and found that these networks were comparable in quality with those provided by the STRING database. Furthermore, we showed that first-in-species gene networks assembled by BiomeNet could effectively retrieve genes annotated for the same AgriGO biological processes. We also demonstrated feasibility of candidate gene enrichment by BiomeNet-based gene prioritization on an example of drought responses in green foxtail, a grass species with sequenced genome for which a functional network model had not been yet available.

## 2 Materials and methods

### 2.1 Network construction using interologs from 95 co-functional source networks

BiomeNet builds a network of user-input proteins by transferring orthologous interactions (also known as interologs) (Yu *et al.*, 2004) from co-functional network sources. Therefore, the quality of the newly constructed networks is largely determined by the accuracy and completeness of the source networks. The current version of BiomeNet contains a total of 95 source networks for 18 species (Supplementary Table S1): 5 animal species, including human (Hwang *et al.*, 2019), mouse (Kim *et al.*, 2016), worm (Cho *et al.*, 2014), zebra fish (Shim *et al.*, 2016) and fly (Shin *et al.*, 2015), six plant species, including Arabidopsis (Lee *et al.*, 2015b), rice (Lee *et al.*, 2015a), soybean (Kim *et al.*, 2017a), tomato ([Bibr btz776-B17][Bibr btz776-B18]), maize ([Bibr btz776-B26][Bibr btz776-B27]) and barley (unpublished), five bacterial species, including *Escherichia coli* ([Bibr btz776-B14][Bibr btz776-B15]), *Klebsiella pneumoniae* (Lee *et al.*, 2019a, b), *Pseudomonas aeruginosa* (Hwang *et al.*, 2016), *Staphylococcus aureus* (Kim *et al.*, 2018), *Xanthomonas oryzae* pv. Oryzae ([Bibr btz776-B20][Bibr btz776-B21]) and two fungal species such as *Cryptococcus neoformans* ([Bibr btz776-B14][Bibr btz776-B15]) and *Saccharomyces cerevisiae* (Kim *et al.*, 2014). Individual networks were inferred from distinct types of omics data (Shim *et al.*, 2017), including: (i) co-citation of two genes in PubMed articles, (ii) co-expression of two genes in various conditions, (iii) associations by domain profiles of protein sequences (Shim *et al.*, 2019; Shim and Lee, 2016), (iv) associations by phylogenetic profiles (Shin and Lee, 2015, 2017), (v) associations by gene neighborhood (Kim and Lee, 2017; Shin *et al.*, 2014), (vi) protein–protein interactions determined by high-throughput experimental assays, collected from literature, or inferred from protein tertiary structure (Aloy and Russell, 2003) and (viii) association by genetic interaction profiles (Kim *et al.*, 2019a). We suggest the following default selection of source networks because it generally enables to achieve the best trade-off between model quality and computational efficiency: use all 95 source networks for building plant gene networks; use animal, bacterial and fungal source networks for building animal or fungal gene networks and use exclusively bacterial source network for building bacterial gene networks. We suggested to include source networks for animals in building plant gene networks, because functional associations between orthologous proteins in animal species were proven useful to complement the lack of those known for plant species (Lee *et al.*, 2010, [Bibr btz776-B26][Bibr btz776-B27]). Rich information of functional associations between proteins conserved in single-cell eukaryotic fungal species generally improve the quality of gene networks for both animal and plant species. Since not many orthologous proteins exist between prokaryotes and eukaryotes, we suggested not to use animal and plant gene networks in building bacterial gene networks for computing efficiency. However, users may select and use any set of source networks for their own network construction.

### 2.2 Fast identification of orthologous functional interactions

Because molecular functions for cellular processes are evolutionarily conserved at the protein sequence level, BiomeNet uses orthology relationship between proteins for mapping interologs. After protein sequences of target species are uploaded by the user, the BiomeNet server starts searching for homologous proteins among the selected source species. As more than one protein isomer can be translated from a single gene, it is desirable to reduce the complexity of functional hypotheses generated from the final network models. To this purpose, we recommend using one protein sequence per gene by modifying the FASTA file to contain a single protein sequence (typically the longest isoform) for each gene. BiomeNet website provides a Python code that can select the longest isoform protein for each gene of the input FASTA file with a given gene and isoform cross reference table. However, this preprocessing of input file is not mandatory for running BiomeNet server. For the identification of homologous proteins, BiomeNet uses a fast protein alignment algorithm DIAMOND (v. 0.8.24) with sensitive mode (Buchfink *et al.*, 2015), because DIAMOND-sensitive operates about 2000 times faster than BLAST with comparable sensitivity. To further accelerate homology mapping, the BiomeNet server distributes search jobs for different source networks into individual central processing unit cores.

We applied BiomeNet for conducting ortholog identification based on bidirectional best hit (BBH), which allowed identification of a pair of orthologous proteins with the highest sequence similarity among proteins encoded by a respective genome. Although the inclusion of co-orthologs (in-paralogs) which were duplicated after speciation is known to improve the sensitivity of analysis in animals and plants (Dalquen and Dessimoz, 2013), we found that the effect was marginal for species with many available source networks. In addition, the identification of co-orthologs was shown to require hours for computing statistical significance with bootstrapping sampling (Remm *et al.*, 2001). Therefore, BiomeNet uses orthologs identified by BBH with DIAMOND.

### 2.3 Network scores and integration

All source networks used by the BiomeNet server are pre-scored by the log likelihood score (LLS) (Lee *et al.*, 2004), which corresponds to the logarithmic likelihood of the hypothesis that two connected genes belonged to the same biological process. These scores are based on gold-standard co-functional gene pairs generally compiled from manually curated annotations for pathways or biological processes in each source species. It may not be possible to rescore the network for a target species using gold-standard co-functional gene pairs, because in general, manually curated pathway annotations are not available for the target species. However, it is reasonable to consider the original likelihood score derived from source networks in the interpretation and integration of networks for the target species because the quality of manually curated pathway annotations is not likely to significantly differ among species.

Since BiomeNet transfers functional interactions from multiple source networks, identical interactions can be supported by many sources with different likelihood scores. The likelihood scores from multiple sources may be efficiently integrated assuming either complete independence or complete correlation among the network sources. The assumption of complete independence allows naïve Bayes integration, in which LLSs from multiple sources can be simply summed up. In contrast, the assumption of complete correlation allows integration of multiple scores by selecting only one with the highest likelihood. We compared the two approaches of data integration with benchmarking of the integrated networks based on the probability of finding connected genes within the same AgriGO biological processes (Tian *et al.*, 2017) which are independent from modeling any of the evaluated networks, and found that integration by taking the highest score generally resulted in better performance. Therefore, BiomeNet integrates scores from multiple source networks using the interaction with the highest likelihood score.

## 3 Results

### 3.1 Networks constructed by BiomeNet and the STRING database have comparable quality

**Fig. 2.**
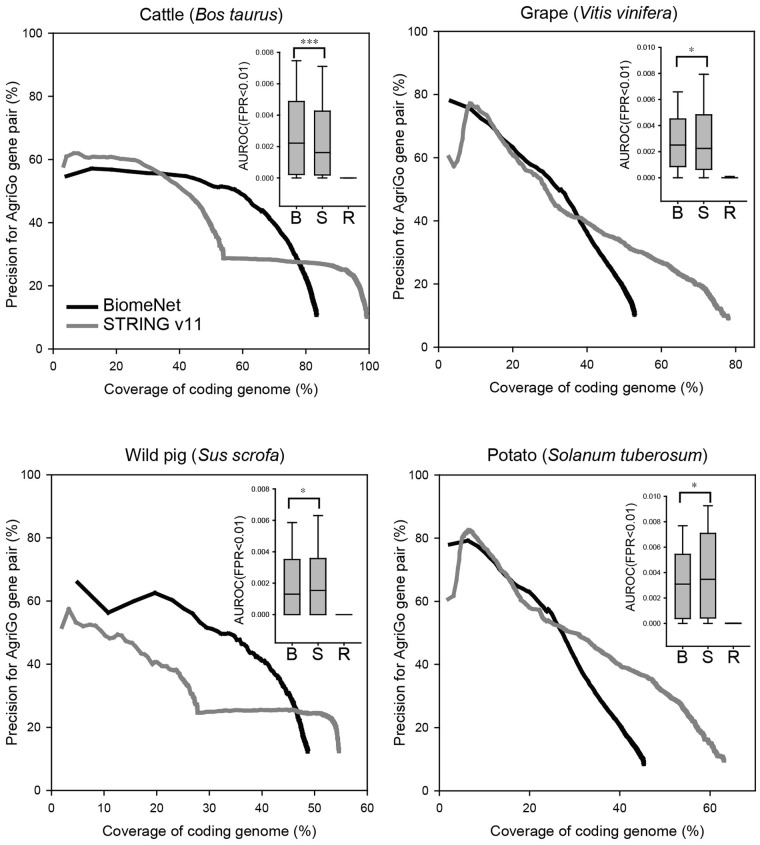
Comparison of networks constructed using BiomeNet and STRING based on prediction of AgriGO annotation. Quality assessment of networks assembled by BiomeNet and STRING was based on the probability of finding two co-functional genes within the same agriGO biological process terms and genome coverage. Predictive powers of the networks for agriGO biological processes were also measured by the AUROC until retrieving 1% of false positive results (FPR < 0.01). BiomeNet appeared to be significantly more predictive than STRING for cattle and grape, whereas STRING was significantly more predictive than BiomeNet for wild pig and potato (*P <* *0.01, **P <* *0.001 and ***P <* *0.0001; Wilcoxon signed rank test). B, S and R of inset represent BiomeNet, STRING and random network, respectively

BiomeNet builds gene networks for target species via interolog-based network transfer (Yu *et al.*, 2004). As it is a relatively simple method of network inference, we compared the quality of networks constructed by BiomeNet and STRING v11 (Szklarczyk *et al.*, 2019), which performs data training and integration for each species. The evaluation was based on the probability of finding connected genes within the same AgriGO biological processes (Tian *et al.*, 2017). For the comparison between BiomeNet- and STRING-constructed networks, we chose two animal and two plant species that have gene networks by STRING and AgriGO biological process annotations: cattle (*Bos taurus*), grape (*Vitis vinifera*), wild pig (*Sus scrofa*) and potato (*Solanum tuberosum*). Analysis of gene networks for the four species constructed by BiomeNet and STRING revealed their comparable quality (Fig. 2). We also assessed the ability of the networks to retrieve genes for the same AgriGO biological processes based on the area under the receiver operating characteristic curve (AUROC) until recall of 1% of false positive results [false positive rate (FPR) < 0.01]. The data indicated that networks constructed by BiomeNet were significantly better in predicting AgriGO biological processes in cattle and grape than those built by STRING (*P *<* *0.0001 and *P *<* *0.01, respectively, by Wilcoxon signed rank test; Fig. 2, inset). These data suggest that the networks automatically constructed by the BiomeNet server can provide the quality and predictive power for biological processes comparable to those constructed by STRING based on training genomics data for each species.

### 3.2 First-in-species gene networks built by BiomeNet are highly predictive for biological processes in animals and plants

**Fig. 3.**
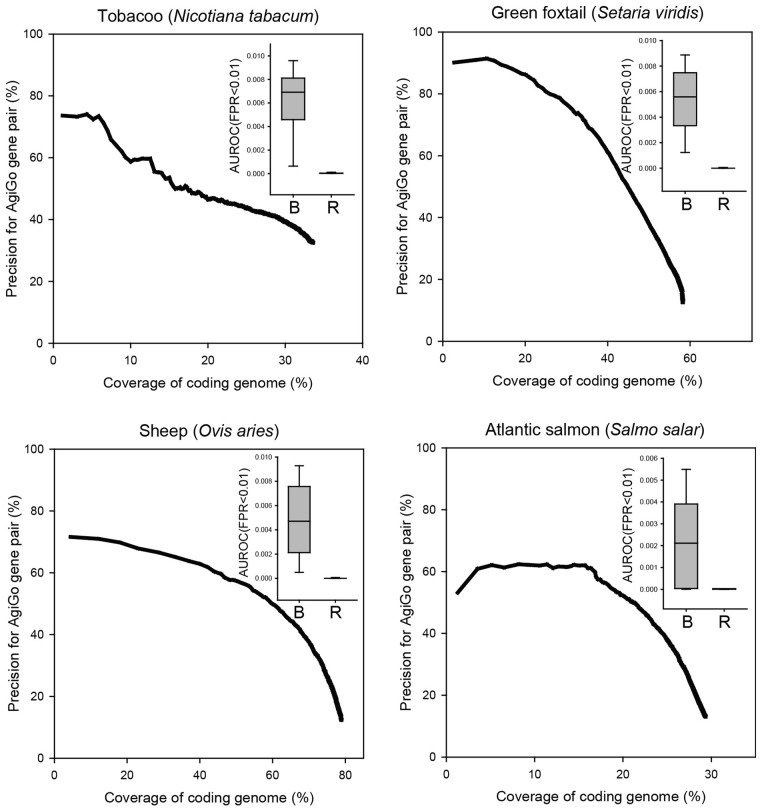
Benchmarking of first-in-species networks by BiomeNet with AgriGO annotation. Quality assessment of networks first constructed for each species by BiomeNet was performed as described for Figure 2

Considering the comparable performance of BiomeNet- and STRING-constructed networks, we expected a reasonable quality of networks for species not yet featured in STRING. Although STRING is the most comprehensive network database, it contains information for only ≤ 10% of all eukaryotic species with sequenced genomes; therefore, the real benefit provided by BiomeNet may be the prediction of gene networks for animals and plants not yet featured in public network databases. We tested the quality of first-in-species gene networks constructed by BiomeNet for tobacco (*Nicotiana tabacum*), green foxtail (*Setaria viridis*), sheep (*Ovis aries*) and Atlantic salmon (*Salmo salar*), which have AgriGO biological process annotations and found that BiomeNet could construct gene networks covering a large proportion of protein-coding genes in these species (33.6%, 58.2%, 78.9%, 29.3%, respectively; Fig. 3). Benchmarking analysis of the ability to retrieve genes for AgriGO biological processes using AUROC (FPR < 0.01) indicated that the gene networks built by BiomeNet had a substantially higher predictive power compared to those assembled by random chance (Fig. 3, inset). These findings suggest that BiomeNet can provide highly predictive gene networks for any species with the sequenced genome.

**Fig. 4.**
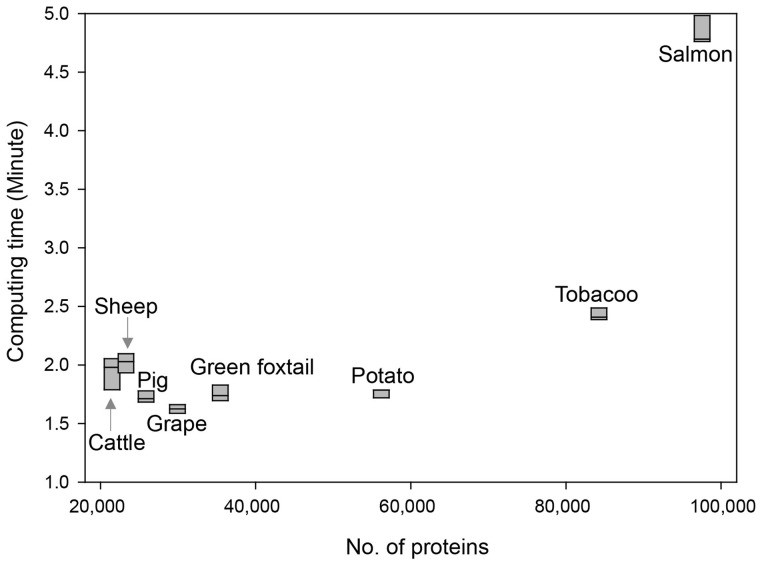
Computing time required to build gene networks for target species with various numbers of input proteins. Computing time was defined as the time period from job submission to return of the constructed network. Analysis was repeated 5 times using all 95 source networks for each of the 8 tested species. Box plots show maximum, median and minimum values from five measurements

During the study, we obtained gene networks for four animal species and four plant species with the number of genes from 22 118 (cattle) to 97 555 (Atlantic salmon). The results revealed that the BiomeNet server could build a network for species with less than 60 000 genes in 2 min and for that with 97 555 genes (Atlantic salmon)––in 5 min (Fig. 4), indicating that BiomeNet could perform gene networking for most species within a few minutes.

### 3.3 BiomeNet provides subnetworks for inferred biological processes

**Fig. 5.**
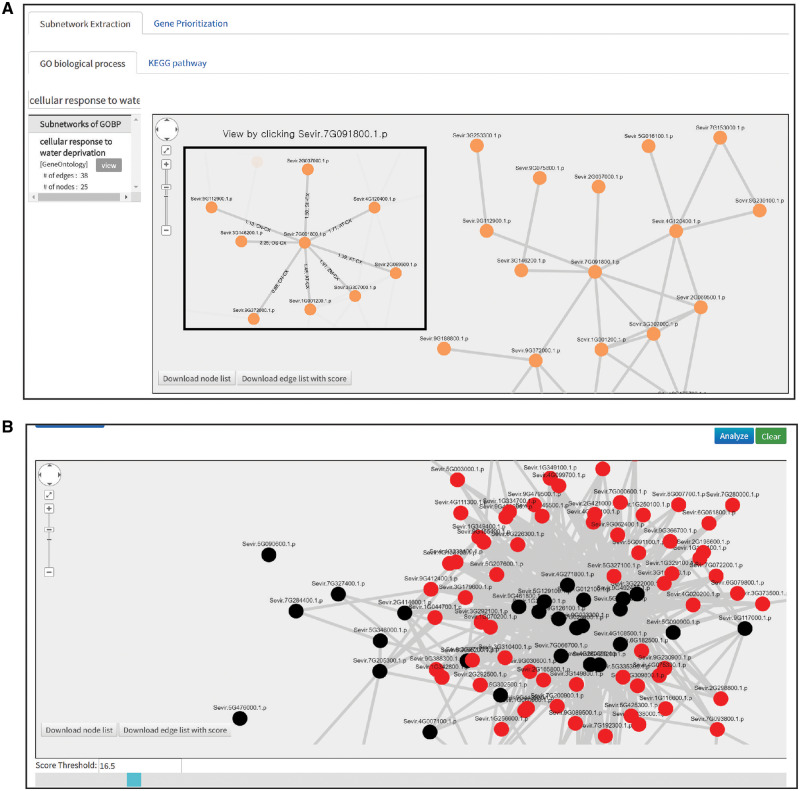
Screenshots of BiomeNet Analyzer pages. (**A**) Subnetwork extraction for inferred GO biological processes and KEGG pathways. Users can search for biological processes of interest with keywords. The visualized network for ‘cellular response to water deprivation’ is composed of 25 nodes and 38 edges. If users click on a particular gene (e.g. Sevir.7G091800.1.p; inset), a network including the selected gene and its neighbors should be highlighted and edge information (source network and score) presented. (**B**) The gene prioritization page displays a network of user-input guide genes (black) and their close neighbors (red). Users can interactively select a threshold (16.5 for the given example) for close neighbors with a range slider

BiomeNet was designed to serve not only for the construction of co-functional gene networks in target species but also as an interface for the users to explore functional modules of interest, which are often represented as subnetworks. To demonstrate the latter feature of BiomeNet, we applied it to listing and visualization of GO-BP subnetworks (The Gene Ontology, 2017) and KEGG pathways (Kanehisa *et al.*, 2017) (Fig. 5A). Newly sequenced genomes may not have annotations in KEGG pathways or GO-BP; therefore, BiomeNet subnetworks are based on inferred KEGG and GO-BP annotations from source species. When BiomeNet transfers functional interactions from source species, it also transfers KEGG and GO-BP annotations associated with orthologous proteins. This approach allows subnetwork extraction for each of inferred KEGG pathways and GO-BP for target species. BiomeNet provides only subnetworks that contain at least three connected genes and visualizes them using Cytoscape.js (Franz *et al.*, 2016). Users can search for KEGG pathways or GO-BP subnetworks with keywords. An example is presented in Figure 5A, which shows a network of genes annotated for the GO-BP term ‘cellular response to water deprivation’, which is composed of 25 nodes (genes) and 38 edges (functional interactions). By clicking on a node and an edge, users may see such information as the likelihood score and supporting evidence, including codes for source species and data type (Fig. 5A inset; also see Supplementary Table S1) and can also obtain lists of member genes and edge information for each subnetwork using a download button inside the network viewer.

### 3.4 BiomeNet-based gene prioritization enables candidate gene enrichment

Another popular application of gene networks is prioritization of candidate genes for pathways or complex traits (Wang and Marcotte, 2010). To test the capability of BiomeNet-based gene prioritization in candidate gene enrichment for complex traits in multicellular organisms, we focused on drought response in *S. viridis*, an emerging model grass species for agronomically important crops such as maize and sorghum (Brutnell *et al.*, 2015). Although the *S. viridis* reference genome has been released (*Setaria viridis* v2.1, DOE-JGI, http://phytozome.jgi.doe.gov/), the genome-scale functional network is not yet available. Using the BiomeNet server, we constructed a network mapping 1 529 150 co-functional links among 20 507 proteins (58.2% of all 35 214 coding genes) within 2 min. Next, we selected 62 *S. viridis* genes upregulated by more than 2-fold in crown roots of plants subjected to drought conditions and harvested 6 days after sowing (Sebastian *et al.*, 2016). In the functional network of *S. viridis* constructed by the BiomeNet server, 51 of the 62 drought response genes were present and well connected to each other (Fig. 5B), suggesting that the obtained network could predict new genes involved in *S. viridis* drought response. The network viewer enabled visual display of the results, as shown on the gene prioritization page which presented a network of user-input proteins (black) and their 100 closest neighbors (red) based on the sum of edge weight scores (i.e. sum of LLSs) for the user-input proteins. Users may also interactively select a threshold score for network neighbors with a range slider.

The user-input genes were ranked based on within-group connectivity by sorting out the genes with the highest sum of edge weight scores. Among the 51 user-input genes, 12 were annotated for inferred GO-BP terms relevant to drought response. Based on the inferred annotations from source species, we found that 367 out of the 35 214 *S. viridis* coding genes were annotated for ‘response to water deprivation’, ‘response to heat’, or ‘heat acclimation’. Considering that the background probability of drought response genes was ∼1% (367/35, 214), it could be concluded that the given transcriptional analysis comparing the response to drought and watered conditions in *S. viridis* crown roots achieved ∼24-fold enrichment for the relevant function. Furthermore, annotation for the involvement in drought response was found for 10 of the top 25 genes (∼40-fold enrichment) but only for 2 of the bottom 26 genes (∼4-fold enrichment), indicating potential utility of the network information for prioritizing candidate genes derived from genomic analysis.

Using the 51 user-input genes as ‘guide genes’, we could prioritize additional candidate genes for drought response based on closeness in the constructed network. BiomeNet provided a table of 200 closest neighbors to the guide genes. We found that 38 of the top 100 candidate genes were associated with drought response (38% discovery rate); among them, four genes encoded proteins related to ‘response to water deprivation’ and 34 of those related to ‘response to heat’ or ‘heat acclimation’. Compared to the background probability of ∼1%, the BiomeNet-based prioritization could achieve ∼38-fold enrichment for relevant biological processes. These results suggest that BiomeNet would facilitate functional annotation of genes involved in complex traits for any species with sequenced genomes.

## 4 Discussion

The most important benefit provided by BiomeNet would be enabling researchers to obtain a gene network for any species with the available genome sequence. The number of eukaryotic species with mapped functional interactome included in the latest issue of STRING, the largest network database, constitutes about 10% of eukaryotes with sequenced genomes. This statistics indicates a considerable gap between genome and interactome information, which may not be reduced unless a computational pipeline allowing automatic network inference for genomes becomes available. In BiomeNet, network inference is based on interologs (Yu *et al.*, 2004) as the server requires information on protein sequences only. Despite of its algorithmic simplicity, BiomeNet provides the quality of networks for AgriGO biological processes comparable with that of STRING. Thus, the results of this study suggest that BiomeNet can be used to construct networks of cellular processes for any sequenced species with reasonable predictive power.

Currently, there are two publicly available web applications for homology-based network inference: the BIANA Interolog Prediction Server (BIPS; http://sbi.imim.es/BIPS.php) (Garcia-Garcia *et al.*, 2012) and JiffyNet (http://www.jiffynet.org) (Kim *et al.*, 2013). Compared to them, BiomeNet offers substantial improvements in several aspects: (i) considerable reduction of computational time (from days to a few minutes), which is especially relevant for animals and plants with tens of thousands of genes; (ii) inferred networks with edge weights (BIPS provides binary interactions only), which are highly useful in downstream analysis and (iii) possibility of conducting functional analysis of the constructed network. With these benefits, BiomeNet represents a homology-based network inference server to be used for network-based functional analysis.

BiomeNet also has limitations. In particular, we observed smaller genome coverage of networks compared to that by STRING, which may be partly due to insufficient use of species-specific data which allow inference of functional links between proteins without interologs in the source species. In the future, we plan to update BiomeNet with additional source networks, which may improve genome coverage of the constructed networks.

As demonstrated by gene prioritization for drought response in *S. viridis*, users can generate new functional hypotheses based on close neighbors to the guide genes in the network. Although in our example of *S. viridis* the guide genes were derived from transcriptomic profiling analysis, they may also be selected based on prior knowledge. Thus, we might have prioritized genes linked to drought response using those with annotation to relevant biological processes such as water deprivation and heat response. Since many GO-BP annotations are associated with complex traits in animals and plants, users will be able to apply BiomeNet-based gene prioritization to studying genetic mechanisms underlying complex traits in newly sequenced species. In conclusion, our results suggest that BiomeNet should enhance the benefit of decoding species genomes for understanding and utilizing biodiversity on Earth.

## Funding

This work was supported by the National Research Foundation of Korea (NRF) grant funded by the Korean Government (MSIT) (NRF-2018M3C9A5064709, NRF-2018R1A5A2025079, NRF-2019M3A9B6065192 to I.L. and NRF-2018M3C9A5064704 to B.L.).

*Conflict of Interest*: none declared.

## Supplementary Material

btz776_Supplementary_Table_1
