# Supplementary material for: BiomeNet: a database for construction and analysis of functional interaction networks for any species with a sequenced genome
Source: Bioinformatics. 2019 Oct 10;36(5):1584–9. doi: 10.1093/bioinformatics/btz776 (PMC7703761; doi:10.1093/bioinformatics/btz776)
Supplement: btz776_Supplementary_Table_1 [file btz776_supplementary_table_1.docx]

**Supplemental Table 1**. List of 95 source networks in BiomeNet database

| **Aminals** | | | | |
| --- | --- | --- | --- | --- |
| ***Homo sapiens* (HS)** | ***Mus musculus* (MM)** | ***Caenorhabditis elegans* (CE)** | ***Danio rerio* (DR)** | ***Drosophila melanogaster* (DM)** |
| Co-citation (CC)  Co-essentiality (CE) |  |  | Co-citation (CC) | Co-citation (CC) |
| Co-expression (CX)  Pathway database (DB) | Co-expression (CX) | Co-expression (CX) | Co-expression (CX) | Co-expression (CX) |
| Domain profile similarity (DP) |  |  |  | Domain profile similarity (DP) |
| Gene neighborhood (GN) | Gene neighborhood (GN) | Gene neighborhood (GN) | Gene neighborhood (GN) | Gene neighborhood (GN) |
| High-throughput PPI (HT) |  | Genetic interaction (GT)  High-throughput PPI (HT) |  | High-throughput PPI (HT) |
| Literature-curated PPI (LC) | Literature-curated PPI (LC) | Literature-curated PPI (LC) | Literature-curated PPI (LC) | Literature-curated PPI (LC) |
| Phylogenetic profile similarity (PG) | Phylogenetic profile similarity (PG) | Phylogenetic profile similarity (PG) | Phylogenetic profile similarity (PG) | Phylogenetic profile similarity (PG) |

| **Plants** | | | | | |
| --- | --- | --- | --- | --- | --- |
| ***Arabidopsis thaliana* (AT)** | ***Oryza sativa* (OS*)*** | ***Glycine max* (GM)** | ***Solanum lycopersicum* (SL)** | ***Zea mays* (ZM)** | ***Hordeum vulgare* (HV)** |
| Co-citation (CC) |  |  |  |  |  |
| Co-expression (CX) | Co-expression (CX) | Co-expression (CX) | Co-expression (CX) | Co-expression (CX) | Co-expression (CX) |
| Domain profile similarity (DP) |  |  |  | Domain profile similarity (DP) | Domain profile similarity (DP) |
| Gene neighborhood (GN) | Gene neighborhood (GN) | Gene neighborhood  (GT) | Gene neighborhood (GN) | Gene neighborhood (GN) | Gene neighborhood (GN) |
| High-throughput PPI (HT) |  |  |  |  |  |
| Literature-curated PPI (LC) | Literature-curated PPI (LC) |  |  |  |  |
| Phylogenetic profile similarity (PG) | Phylogenetic profile similarity (PG) | Phylogenetic profile similarity (PG) | Phylogenetic profile similarity (PG) | Phylogenetic profile similarity (PG) | Phylogenetic profile similarity (PG) |

| **Bacteria** | | | | |
| --- | --- | --- | --- | --- |
| ***Escherichia coli* (EC)** | ***Klebsiella pneumoniae* (KP)** | ***Pseudomonas aeruginosa* (PA)** | ***Staphylococcus aureus* (SA)** | ***Xanthomonas oryzae pv. oryzae* (XO)** |
| Co-citation (CC) | Co-citation (CC) | Co-citation (CC) |  |  |
| Co-expression (CX) |  | Co-expression (CX) | Co-expression (CX) | Co-expression (CX) |
| Domain profile similarity (DP) | Domain profile similarity (DP) | Domain profile similarity (DP) | Domain profile similarity (DP) | Domain profile similarity (DP) |
| Gene neighborhood (GN) | Gene neighborhood (GN) | Gene neighborhood (GN) | Gene neighborhood (GN) | Gene neighborhood (GN) |
| High-throughput PPI (HT) |  |  | High-throughput PPI (HT) |  |
| Literature-curated PPI (LC) |  |  |  |  |
| Phylogenetic profile similarity (PG) | Phylogenetic profile similarity (PG) | Phylogenetic profile similarity (PG) | Phylogenetic profile similarity (PG) | Phylogenetic profile similarity (PG) |

| **Fungi** | |
| --- | --- |
| ***Cryptococus neoformans* (CN)** | ***Saccharomyces cerevisiae* (SC)** |
| Co-citation (CC) | Co-citation (CC) |
| Co-expression (CX) | Co-expression (CX) |
| Domain profile similarity (DP) | Domain profile similarity (DP) |
| Gene neighborhood (GN) | Gene neighborhood (GN) |
|  | Genetic interaction (GT) |
|  | High-throughput PPI (HT) |
|  | Literature-curated PPI (LC) |
| Phylogenetic profile similarity (PG) | Phylogenetic profile similarity (PG) |
|  | PPI inferred by 3-D structure (TS) |
